# Author Correction: Boundary effects of expectation in human pain perception

**DOI:** 10.1038/s41598-020-75632-2

**Published:** 2020-11-04

**Authors:** E. J. Hird, C. Charalambous, W. El-Deredy, A. K. P. Jones, D. Talmi

**Affiliations:** 1 Division of Neuroscience and Experimental Psychology, University of Manchester, Manchester, UK; 2 School of Mathematics, University of Manchester, Manchester, UK; 3 Institute of Psychiatry, Psychology and Neuroscience, Kings College London, London, UK; 4 Centro de Investigación Y Desarrollo en Ingeniería en Salud, Universidad de Valparaiso, Valparaiso, Chile; 5 Human Pain Research Group, Salford Royal NHS Foundation Trust, Manchester, UK; 6 Department of Psychology, Downing Site, University of Cambridge, Cambridge, UK

Correction to: *Scientific Reports* 10.1038/s41598-019-45811-x, published online 01 July 2019

The original version of this Article contained errors.

There was a typographical error in the spelling of the author A. K. P. Jones, which was incorrectly given as A. K. Jones.

As a result, the Author Contributions section was incorrect.

“E.J.H.: study conception, data collection, data analysis, paper writing. C.C.: data analysis, paper writing. W.E.D.: study conception, paper writing. A.K.J.: study conception, paper writing. D.T.: study conception, paper writing.”

now reads:

“E.J.H.: study conception, data collection, data analysis, paper writing. C.C.: data analysis, paper writing. W.E.D.: study conception, paper writing. A.K.P.J.: study conception, paper writing. D.T.: study conception, paper writing.”

In addition, there were errors in the affiliation list.

Firstly, an affiliation was omitted for E. J. Hird, W. El-Deredy, A. K. Jones & D. Talmi, which is now given as Affiliation 1.

Secondly, a second affiliation was omitted for D. Talmi, which is now listed as Affiliation 6.

Thirdly, E. J. Hird, W. El-Deredy, A. K. Jones & D. Talmi were incorrectly affiliated with ‘School of Mathematics, University of Manchester, Manchester, UK’.

Finally, Affiliation 5 was incorrectly given as ‘Salford Royal NHS Foundation Trust, Manchester, UK’.

The correct affiliations are listed below:

Affiliation 1:

Division of Neuroscience and Experimental Psychology, University of Manchester, Manchester, UK

Affiliation 2:

School of Mathematics, University of Manchester, Manchester, UK

Affiliation 3:

Institute of Psychiatry, Psychology and Neuroscience, Kings College London, London, UK

Affiliation 4:

Centro de Investigación y Desarrollo en Ingeniería en Salud, Universidad de Valparaiso, Valparaiso, Chile

Affiliation 5:

Human Pain Research Group, Salford Royal NHS Foundation Trust, Manchester, UK

Affiliation 6:

Department of Psychology, University of Cambridge, Downing Site, Cambridge, UK

These errors have now been corrected in the PDF and HTML versions of the Article, and in the accompanying Supplementary Information file.

